# The Frequency of Aberrant CD7 Antigen Expression in Acute Myeloid Leukaemia Patients

**DOI:** 10.7759/cureus.22309

**Published:** 2022-02-16

**Authors:** Hassan Raza, Mavra Fatima, Tayyab Noor, Shereen Umer, Ayisha Imran, Nauman A Malik

**Affiliations:** 1 Hematology, Chughtai Lab, Lahore, Lahore, PAK

**Keywords:** acute myeloid leukemia, blast cells, flowcytometry, cd7, aberrant expression

## Abstract

Background

Aberrant phenotype expression in acute myeloid leukemia (AML) may be due to genetic defects and is associated with a poor prognosis. CD7 is the first T-cell-associated antigen to be expressed during T-lymphocyte maturation. Aberrant expression of CD7 in AML influences clinical response, remission rate, and overall survival in these patients.

Objective

To determine the frequency of aberrant CD7 expression in patients with AML.

Materials and methods

This cross-sectional study was performed over a period of 12 months from July 2020 to June 2021 in the Hematology Department, Chughtai Lab, Lahore. This study included 120 patients who were newly diagnosed with AML. The following tests were performed for included patients: complete blood count (CBC), peripheral blood smear analysis, and flow cytometric analysis using a blood sample or bone marrow aspirate. Blast cells were analyzed for aberrant CD7 expression. Calculation of the sample size was performed by using the Select Statistics calculator. All statistical analyses were performed using SPSS ver. 23 software (IBM Corp., Armonk, NY). Data were expressed as frequencies, means ± standard deviation (SD), and percentages.

Results

Of 120 patients newly diagnosed with AML, the CD7 antigen was aberrantly expressed in 36 cases (30%). Of these patients, the AML2 subtype was the most common type of AML with aberrant CD7 expression, followed by AML M4, AML M1, M3, AML M5, and AML M0, respectively.

Conclusion

In our study, aberrant CD7 expression occurred at a high frequency in acute myeloid leukemia. Thus, this marker should be added to the current flow cytometry panels.

## Introduction

Acute leukemias comprise a category of cancers that share clinical, morphologic, immunologic, and molecular characteristics as well as surface antigen (CD antigen) expression patterns. Many of the surface molecules expressed by blast cells in acute leukemia are known as cluster of differentiation (CD) antigens [1]. Every kind of leukemia expresses specific CD markers, which are currently used to classify hematological malignancies and serve as the foundation for diagnosis. Flow cytometry can be used to assess the expression of CD markers. Acute leukemia categorization becomes more accurate because of immunophenotyping [2].

In South Asia, acute lymphoblastic leukemia (ALL) and acute myeloid leukemia (AML) account for approximately one-third and one-fifth of all hematological malignancies, respectively [3]. According to previous studies, AML is the most prevalent type of leukemia in Pakistan, whereas AML is the second most common type of leukemia in the United States. The incidence of these cancers is among the top 10 causes of death in developing countries [4]. Acute myeloid leukemia is defined as the presence of 20% or more blast cells in the bone marrow and is the most prevalent type of acute leukemia in adults. AML has a wide range of phenotypes due to cytogenetic and molecular alterations. Several studies have found that T-cell antigens are abnormally expressed in patients with AML. Aberrant expression of these antigens occurs when cells display aberrant proteins or when they lack expression of markers specific to that cell type [5].

CD7 is a T-cell antigen that is expressed in a small percentage of individuals with AML. Somatic cell hybridization was used to localize the CD7 gene to chromosome 17 [6]. In most studies, CD7 is the most prevalent aberrant marker in AML. CD7 expression has been linked to the first stages of myeloid differentiation, and many studies have shown that CD7 expression should not be regarded as a T-lineage-specific antigen because it is frequently observed in individuals with AML. In these patients, aberrant CD7 expression affects clinical response, remission rate, and overall survival [7,8]. Since there is a lack of relevant data in Pakistan, our study aimed to determine the frequency of aberrant CD7 marker expression in patients with AML.

## Materials and methods

This study included 120 patients who were newly diagnosed with acute myeloid leukemia. Patients were labeled with acute myeloid leukemia based on peripheral blood smears, bone marrow aspirates, and flow cytometry. Those showing 20% blast cells in peripheral blood or bone marrow aspirate and positive CD markers of CD34, MPO, CD13, CD33, CD117 and negative for Tdt, CD19, CD79a, and CD20 were labelled as acute myeloid leukemia. The patients were recruited to the Chughtai Lab in Lahore for a period of one year (July 2020-June 2021). The sample size was calculated by using the Select Statistics sample size calculator (http://select-statistics.co.uk/calculators/) where the confidence level was 95% and the margin of error was 5.5%. Patients diagnosed with AML having blast cells showing CD7 antigen expression greater than 20% were included in the study. Patients with ALL, mixed phenotype acute leukemia (MPAL), patients on treatment, having blast percentages of less than 20%, and those having blast cells showing less than 20% aberrant CD7 expression were excluded from the study.

The study was approved by the institutional review board of our institution (CIP-IRB-1066). Following informed consent, the medical history of the patients was obtained, and clinical examinations were conducted. Additionally, they underwent the following tests: complete blood count (CBC), peripheral blood smear, and flow cytometric analysis of peripheral blood samples or bone marrow aspirate. For CBC and bone marrow aspirate, 1 ml of blood was taken from each patient and dispensed in EDTA viol. Blood samples were derived from either peripheral blood or bone marrow aspirate depending on the availability of blast cells and the required percentage. All peripheral blood or bone marrow aspirate smears were air-dried and subsequently stained with Leishman stain for microscopy analysis. All peripheral blood smears or bone marrow aspirates were stained with Sudan Black B.

Immunophenotyping was performed on the BD FACSCalibur™ flow cytometry system. Two milliliters of peripheral blood or bone marrow aspirate were obtained. Blast cells were gated using forward and side scatters and analyzed using cell quest-Pro software (BD Biosciences, San Jose, CA). Fresh peripheral or bone marrow samples were kept at ambient temperature and processed for immunophenotyping within six hours of collection. The aberrant CD7 expression on myeloblasts was then analyzed. Antigen expression was considered positive when the percentage of blast cells was equal to or greater than 20. Similarly, aberrant expression was considered positive when 20% of blast cells expressed aberrant CD7. All statistical analyses were performed using SPSS ver. 23 software. Data were expressed as frequencies, means ± SD, and percentages.

## Results

In this cross-sectional study, 120 newly diagnosed AML patients were enrolled to determine the expression of aberrant CD7 in diagnosed cases of AML. In our study, the mean age of the patients was 41.3 ± 18.7 years. Details of patients of various age groups are shown in Table 1. The majority of cases were older than 50 (Table 1). The frequency of AML was higher in males than in females, and the male-to-female ratio was 2:1 (Table 2). Abnormal CD7 antigen expression in relation to age is shown in Table 3. The maximum number of (13 cases) falls in the age group greater than 50 years. Aberrant expression via flow cytometry was observed in 30% of patients. AML2 was the most common AML type with aberrant CD7 expression, followed by AML M4, AML M1, AML 2, M3, AML M5, and AML M03. This aberrant expression was observed in certain (French-American-British) FAB subtypes of AML in our study, as shown in Table 4.

**Table 1:** Distribution of age in all AML patients AML: acute myeloid leukemia

Age in years	No. of patients included (n= 120)	Percentage
<10 years	6	5
11-20 years	11	9
21-35 years	25	21
36-50 years	35	29
More than 50	43	36

**Table 2:** Distribution of all AML FAB subtypes in relation to gender AML: acute myeloid leukemia, FAB: French-American-British, M0: acute myeloid leukemia with minimal maturation, M1: acute myeloid leukemia without maturation, M2: acute myeloid leukemia with maturation, M3: acute promyelocytic leukemia, M4: acute myelomonocytic leukemia, M5: acute monocytic/monoblastic leukemia.

FAB classification	Males	Females
M0	7	5
M1	13	7
M2	6	7
M3	18	8
M4	14	5
M5	14	16
Total	72	48

**Table 3:** Distribution of age regarding aberrant CD7 expression (n=120) AML: acute myeloid leukemia, FAB: French-American-British, M0: acute myeloid leukemia with minimal maturation, M1: acute myeloid leukemia without maturation, M2: acute myeloid leukemia with maturation, M3: acute promyelocytic leukemia, M4: acute myelomonocytic leukemia, M5: acute monocytic/monoblastic leukemia.

	CD7 expression	
Age group	Yes	No
<10 years	1	5
11-20 years	4	7
21-35 years	10	15
36-50 years	8	27
More than 50	13	39
Total	36	84

**Table 4:** Frequency of AML cases with aberrant CD7 expression among various FAB classes of AML AML: acute myeloid leukemia, FAB: French-American-British, M0: acute myeloid leukemia with minimal maturation, M1: acute myeloid leukemia without maturation, M2: acute myeloid leukemia with maturation, M3: acute promyelocytic leukemia, M4: acute myelomonocytic leukemia, M5: acute monocytic/monoblastic leukemia.

AML subtype (FAB classification)	AML patients	CD7 expression
AML M0	12	3
AML M1	20	7
AML M2	13	9
AML M3	26	5
AML M4	19	7
AML M5	30	5
Total	120	36

## Discussion

Antigen expression on neoplastic cells that varies from the expression during the normal hematological maturation process is referred to as an aberrant phenotype in acute leukemia. Lineage infidelity (lymphoid marker expression in myeloid blast cells) and asynchronous antigen expression are two examples of aberrant antigen expression in acute leukemia [6]. The aberrant expression occurs when cells express aberrant markers or when they lack lineage markers that are not linked with that particular cell type. During T-lymphocyte maturation, CD7 is the first T-cell-associated antigen to be expressed [7]. In patients with AML, aberrant expression of the CD7 marker influences clinical response, remission rate, and overall survival. Studies have shown that aberrant phenotypes are present in most patients with AML. Flow cytometry can be used to detect aberrant antigen expression in lymphoma/leukemia cells and can aid in determining the origin of the cells [8].

In our study, the average age of the patients was 41.3 ± 18.7 SD years. Details of the patients in the various age groups are shown in Table 1. The incidence of AML was higher in males than in females, and the male-to-female ratio was 2:1. Most patients were older than 40 years of age. Aberrant expression of CD7 was observed in 30% of patients via flow cytometry. This aberrant expression was seen in different FAB subtypes in our study, as shown in Table 3. Many studies have shown that aberrant CD7 is expressed in acute myeloid leukemia markers at various frequencies, ranging from 3% to 42%. Some studies have demonstrated that CD7 is expressed in more immature subtypes. This was explained according to the notion that the CD7 antigen is transiently expressed during the very early stages of myeloid progenitor differentiation, after which its expression is downregulated as myeloid progenitors undergo further differentiation and maturation.

The most prevalent markers observed were CD7 and TdT, which were expressed in 44.4% of the individuals examined. Notably, CD7 was identified in 40% of AML patients with poorly differentiated cancer, whereas TdT was found in 44%. Our study showed aberrant expression in 30% of cases [9]. In another study [10], CD7 expression was observed in 15 of 46 patients with AML (32.6%). Eight of 10 patients with AML expressed CD7 expression, whereas four exhibited CD79a positivity. Hence, in the less-differentiated AML subtypes, such as FAB M0, M1, and M2, these markers are expressed early in hemopoietic ontogeny [10]. Similar to this study, CD7 expression in M5 was 30.7% and in MO it was 25%.

Clinically, the identification of aberrant phenotypes is critical not only for accurate diagnosis but also for AML and ALL subclassification. In one study, 30 cases of AML were tested for CD2, CD3, CD5, CD7, CD8, and TdT. The authors hypothesized that CD7 was commonly expressed at higher levels and that TdT was present in more than one-third of the cases. Expression of the antigen CD3, which is unique to the T-cell lineage, was absent [11]. In a local study conducted in Pakistan, flow cytometry was utilized to immunophenotype 50 patients with AML. AML was diagnosed in 23 of the cases, whereas ALL was diagnosed in 27. CD7 expression was detected in six (26%) of 23 AML patients, whereas CD5 positivity was observed in three individuals (13%). Tdt expression was demonstrated in five (22%) of the patients with AML. It was concluded that leukemia cells express atypical markers as a result of their defective genetic program, which causes them to exhibit aberrant immunophenotypes [12].

In another study, all cases of acute leukemia were studied: 53 cases of AML, 43 cases of ALL, and four cases of MPAL were identified from a total of 100 patients with suspected leukemia. ALL was divided into B-ALL and T-ALL on the basis of immunophenotyping, and B-ALL accounted for 88.4%, whereas T-ALL accounted for 11.6% of total ALL cases (43.0%). CD7, CD33, and CD13 were the most frequently expressed antigens in AML, with CD7 being the most prevalent abnormality [13]. In a study from Bangladesh, Rashid et al. reported 64 patients with acute leukemia, including 31 cases of AML, 21 cases of B-ALL, and 10 cases of T-ALL. MPAL was identified in the remaining two cases (3.1%). Of these patients, 40.3% (25/62) exhibited aberrant expression of CD markers. Seven (22.5%) of the 31 AML patients with AML had aberrant CD7 expression, followed by CD19 (12.9%) in four and TdT (3.2%) in one [14].

In another report, Hamid and Akrabi documented a total of 55 acute leukemia cases, and of these, 29 (52.7%) were acute lymphoblastic leukemias and 26 (47.3%) were AML. All AML subtypes were represented, and one case was diagnosed with AML with a mixed phenotype according to flow cytometry (biphenotypic). Patients with AML (43%) and ALL (32%) showed aberrant immunophenotype expression. CD19 and CD7, which were the most common aberrant lymphoid markers detected in AML, were observed in 30.8% and 26.9% of cases, respectively. CD4 expression was aberrant in 15.4% of patients with AML. CD20 was aberrant in 15.4%, CD79a was aberrant in 10.5%, and CD10 was aberrant in 10.5% [15].

In one study, a FACSCaliburTM flow cytometer was used to analyze 1 ml samples of peripheral blood or bone marrow aspirate. CD13 and CD33 were the most frequently expressed aberrant markers in patients with ALL, whereas CD7 and CD19 were the most frequently expressed aberrant markers in patients with acute myeloid leukemia. CD5, CD7, CD64dim, CD10, CD117, CD25, and TdT were expressed in 32% (9/28) of AML patients [16]. In a retrospective analysis of data from 144 patients with AL, it was discovered that 61.8% of patients with AL had AML and that AML-M2 (31.8%) was the most prevalent FAB subtype in the AML group, followed by M4-M5 (27.3%). CD7 was the most frequently expressed lymphoid antigen in AML (25%) [17].

In another study, 181 newly diagnosed AML cases were included. The age range of the patients was wide (range 1-81 years; median 30 years), with a male-to-female ratio of 1.42:1. Adults (range 17-81 years of age; median 40 years) accounted for 135 of the total AML cases, with a male-to-female ratio of 1.1:1. Forty-six cases were in the pediatric age category, with a male-to-female ratio of 1.23:1. In all, 43.1% of AML patients expressed aberrant lymphoid antigens. The aberrant expression of T-cell lineage-related markers (CD7/CD4/CD8) was the most prevalent and accounted for 73% (57/78) of all aberrancies detected. CD7 was the most prevalent aberrant lymphoid antigen, as it was present in 26.5% of all cases and was more frequent in pediatric AML patients (28.2% vs. 25.9% of adult AML cases) [18]. All FAB subtypes except AML-M3 showed aberrant lymphoid characteristics. AML-M4 had the highest rate of expression at 76%, followed by AML-M0 and AML-M1 at 62.5% each and AML-M2 at 41.6%. Other studies reported that aberrant lymphoid markers were not a common finding in AML-M3 [19,20].

Leukemia is diagnosed and classified according to a combination of morphology, cytochemistry, flow cytometry, and cytogenetics. Flow cytometry is quite useful for the diagnosis of AL, especially in ALL for lineage assignment and classification of MPAL. Flow cytometry also aids in the identification of minimal residual disease by identifying aberrant antigen expression. Some clinicians believe that patients with aberrant CD7 antigen expression exhibit a shorter survival time and are more likely to exhibit non-responsive behavior to the recommended treatment protocol than CD7-negative patients [21]. Further studies should be performed in this regard, including in our local population, to determine the frequency of aberrant antigen expression, including that of CD7, and to establish the impact of CD7 expression on treatment response, recurrence, and overall survival.

Limitations

A number of limitations exist in this study. (1) There was a limited population. (2) The patients' cytogenetics and molecular analysis were not performed. (3) Patients were not followed up to determine the impact of aberrant CD7 on treatment and prognosis. There is a need for further research to investigate the correlation between aberrant CD7 expression, cytogenetics, and other molecular abnormalities in patients with AML.

## Conclusions

In our study, 30% of AML cases showed aberrant CD7 expression, which is in agreement with previous studies. This is a significant finding since aberrant CD7 expression affects the recommendation of treatment regimens, the clinical course, and the disease outcome. It is recommended that additional studies be conducted to observe the relationship between aberrant CD7 expression, cytogenetics, and other molecular abnormalities in patients with AML and to determine whether CD7 antigen expression influences survival, recurrence, and treatment response.
